# 

**Published:** 1866-02

**Authors:** 


### CONTENTS.

### ORIGINAL CONTRIBUTIONS.

ART. III. A Case of Ovariotomy. By E. B. Wolcott, M.D., Milwaukee, Wis., ---- 65
ART. IV. Blood-letting not Necessary in Pneumonia. By D. W. Young, M.D., Aurora, Ill., ---- 68
ART. V. A Paper on Opium in Certain Forms of Fever, and in Diarrhœa and Dysentery. By S. B. Hawley, M.D., Aurora, Ill., 76
ART. VI. Dr. C. F. Taylor's Spinal Apparatus. By F. O. Earle, M.D., New York. ---- 81
ART. VII. Laughing in Laryngoscopy, and a new Mode of Applying Solutions to the Larynx. By H. A. Johnson, M.D., of Chicago, ---- 82
ART. VIII. Muriate of Ammonia in Iritis. By Jos. S. Hildreth, M.D., Chicago, late Surgeon in charge of the Desmarres (U.S.A.) Eye and Ear Hospital, ---- 106

### CORRESPONDENCE.

Letter from Dr. David Prince, ---- 83

### SELECTIONS.

Periscope of Medical and General Science in their Relations to Dentistry. By Geo. J. Ziegler, M.D., ---- 88
The Sale of Poisons in Bombay, ---- 93
On Facial Hemiplegia and Paralysis of the Facial Nerve. By Wm. R. Saunders, M.D., F.R.C.P.Ed., Physician to Royal Infirmary, Edin., 94

### BOOK NOTICES.

Obscure Diseases of the Brain and Mind. By Forbes Winslow, M.D., D.C.L., Oxon., &c., &c., ---- 110
Experimental Investigations into the Action and Uses of the Bromide of Potassium. By Roberts Bartholow, M.D., Prof. of Physics and Medical Chemistry in the Medical College of Ohio, ---- 111
Lectures on the Diseases of Infancy and Childhood. By Charles West, M.D., Fellow of the Royal College of Physicians, &c., ---- 112

### EDITORIAL.

Our State and National Medical Associations, ---- 113
Chicago Medical Society, ---- 113
Case of Cerebro-Spinal Meningitis, ---- 116
Annual Commencement of the Chicago Medical College, ---- 118
Rush Medical College Commencement, ---- 118
London Lancet, ---- 119
American Medical Association, ---- 119
Obituary.—Death of Dr. Wm. B. Herrick, ---- 120
Copaiba Deprived of its Disagreeable Smell, ---- 121
A Nice Point of Differential Diagnosis, ---- 121
The Mortality Statistics for January, 1866, ---- 121
Deaths during the Year 1865, ---- 123
Causes of Death in Chicago in 1865, ---- 123
Deaths in Chicago in Former Years, ---- 125
Another Victim of the Risks connected with the Medical Profession, ---- 125
Vaccination and Syphilis, ---- 125
Galactozyme, ---- 125

